# Light‐Mediated Supramolecular Functionalization of Polymerization‐Induced Self‐Assembled Micelles

**DOI:** 10.1002/smll.202511176

**Published:** 2025-11-06

**Authors:** Ruggero Rossi, Miriam Abad, Luis Oriol, Daniele Martella, Camilla Parmeggiani, Milagros Piñol

**Affiliations:** ^1^ Department of Chemistry “Ugo Schiff” University of Florence via della Lastruccia 3‐13 Sesto Fiorentino 50019 Italy; ^2^ European Laboratory for Non‐Linear Spectroscopy (LENS) via N. Carrara 1 Sesto Fiorentino 50019 Italy; ^3^ Instituto de Nanociencia y Materiales de Aragón (INMA) CSIC‐Universidad de Zaragoza C/ Pedro Cerbuna, 12 Zaragoza 50009 Spain; ^4^ Departamento de Química Orgánica Facultad de Ciencias Universidad de Zaragoza C/ Pedro Cerbuna, 12 Zaragoza 50009 Spain

**Keywords:** light‐response, photoswitches, polymeric nanocarriers, RAFT‐PISA, supramolecular functionalization

## Abstract

Precise, remote control of polymeric nanoparticles via external stimuli is a key aim for the next‐generation drug delivery. As a versatile, one‐pot method, polymerization‐induced self‐assembly (PISA) produces dispersions of block‐copolymer micelles at high solids content with tunable core–corona architectures. Light is an ideal trigger to control the uptake and delivery of specific molecules inside such micelles since it can be localized, and easily tuned in intensity and wavelength. In this study, PISA is employed in water to obtain micelles containing diacylaminopyridine units functionalizable thanks to supramolecular interaction by a light‐mediated process. Indeed, only if UV irradiation is used to convert thymine‐based azo photoswitches into the *cis* form, these molecules permeate the hydrophilic corona and anchor via hydrogen bonds to the hydrophobic core of the micelles. Subsequent visible‑light exposure regenerates their *trans* state without micelle disassembling. The photoswitch loading boosts the encapsulation of Nile Red, studied here as a model of hydrophobic cargo, while a subsequent UV light stimulus accelerates the dye release; moreover, the selected photoswitch sustains release over days without further irradiation. By marrying advanced polymerization techniques with reversible photochemistry, dynamic micelles are prepared whose structure and cargo release can be fully controlled by light.

## Graphical abstract

A one‐pot, light‐controlled approach to “switch on” Polymerization‐Induced Self‐Assembly (PISA) micelles with a novel azo‐photoswitch is introduced. A brief UV pulse converts the photoswitch to its polar *cis* form, driving it into the micelle core and anchoring via hydrogen bonds. A subsequent visible‐light step regenerates the nonpolar *trans* state without disassembly of the micelles. Loaded with Nile Red, these dynamic carriers release cargo on demand and sustain delivery for days after a single light activation.

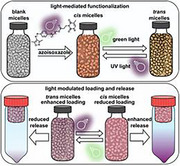

## 1 Introduction

Smart nanocarriers, ranging from liposomes and dendrimers to polymeric micelles, are widely exploited to deliver poorly soluble therapeutics improving their bioavailability and reducing off‐target effects.^[^ ^1^, ^2^, ^3^, ^4^, ^5^, ^6^, ^7^ ^]^ Among them, polymeric micelles self‐assembled from amphiphilic block copolymers have shown particular promise in cancer therapy, as they solubilize hydrophobic drugs and passively target tumors.^[^ ^8^, ^9^, ^10^, ^11^ ^]^ The synthesis of block copolymers can be easily faced using controlled radical polymerizations, which usually offer good compatibility with a wide range of functional monomers. In particular, Reversible Addition‐Fragmentation chain Transfer (RAFT) polymerization, a technique that uses a Chain‐Transfer Agent (CTA) to mediate the reversible equilibrium between growing radicals and dormant chains, provides exceptional control over polymerization degree and polymer dispersity.^[^ ^12^, ^13^ ^]^ Coupling RAFT with Polymerization‐Induced Self‐Assembly (PISA) allows for a precise control over both polymerization and the morphology of the self‐assemblies: as amphiphilic block copolymers form in situ at high monomer concentrations (often over 25 wt%), they spontaneously organize into uniform micelles, worms, or vesicles depending on the conditions.^[^ ^14^, ^15^ ^]^ Therefore, the one‐pot RAFT‐PISA approach combines scalable synthesis with finely tuned architectures and surface functionalities, making it an ideal platform for targeted drug delivery and other advanced biomedical applications.^[^ ^16^ ^]^ Other methods, like microfluidics, can produce extremely uniform particles but only at low flow rates and with specialized equipment.^[^ ^17^ ^]^ Emulsification or template‐based techniques can also shape particles but often rely on surfactants, multiple cleanup steps, or harsh solvents.^[^ ^18^, ^19^ ^]^ In contrast, PISA can assemble carriers directly in water under mild conditions, combining scale, precision, and simplicity in one straightforward process.^[^ ^20^ ^]^

A key challenge in drug delivery is avoiding systems that release their entire payload in a short burst, leading to an initial concentration spike followed by levels that rapidly fall below therapeutic efficacy. Many conventional nanocarriers suffer from this uncontrolled release, which necessitates frequent re‐administration, increases toxicity, and adds to patient burden.^[^ ^21^, ^22^ ^]^ As an alternative, smart nanocarriers can respond to specific stimuli to release drugs at controlled rates and targeted sites. Sustained on‐demand delivery maintains therapeutic levels longer, improving efficacy and reducing side effects. Stimuli‐responsive nanocarriers have been engineered to respond to internal or external cues such as pH, redox gradients, temperature, or light.^[^ ^23^, ^24^, ^25^, ^26^, ^27^, ^28^, ^29^, ^30^ ^]^ Although light can offer a precise and reversible modulation on polymer properties combined with wireless control, its poor tissue penetration in biological tissue makes in vivo irradiation difficult for implementing a dynamic control in real applications.^[^ ^31^ ^]^ In contrast, if light is delivered only before the nanocarriers administration to influence the following release, such a control can be cheaper and easier with respect to other stimuli (e.g., through LED lamps). Photoresponsive units, such as azobenzenes and spiropyrans, have been inserted into micelles, vesicles, and nanoworms to leverage by light the disruption of core packing or membrane integrity, triggering cargo release via swelling, poration, or filament reshaping.^[^ ^32^, ^33^, ^34^, ^35^, ^36^, ^37^, ^38^, ^39^ ^]^ Indeed, azo chromophores have been the most used for light‐responsive units, owing to the pronounced differences in the chemical‐physical properties of their *trans* and *cis* isomers.^[^ ^40^ ^]^ The thermodynamically stable *trans* form is nearly planar, elongated, and essentially nonpolar. Upon UV irradiation, it undergoes rapid, robust *trans‐cis* photoisomerization to a bent, nonplanar geometry with a substantially increased dipole moment. This switching is fully reversible and fatigue‐resistant: visible light (or thermal relaxation) returns the molecule to the apolar *trans* state without any loss of performance over repeated cycles.^[^ ^41^ ^]^ Also, azoheteroarene photoswitches (azobenzene derivatives in which one aromatic ring is replaced by a heteroarene, such as arylazoisoxazole, abbreviated as AIZ), are prized for the rapidity and reliability of their *trans–cis* isomerizations and their excellent thermal stability; however, their application in functional nanocarriers and drug delivery systems remains largely unexplored.^[^ ^42^, ^43^, ^44^, ^45^ ^]^ However, direct incorporation of such azo monomers into different radical polymerization processes often suffers from low conversion and broad molecular‐weight distributions, require organic co‐solvents for assembly, and achieve only modest switch and drug loadings.

**Figure 1:**
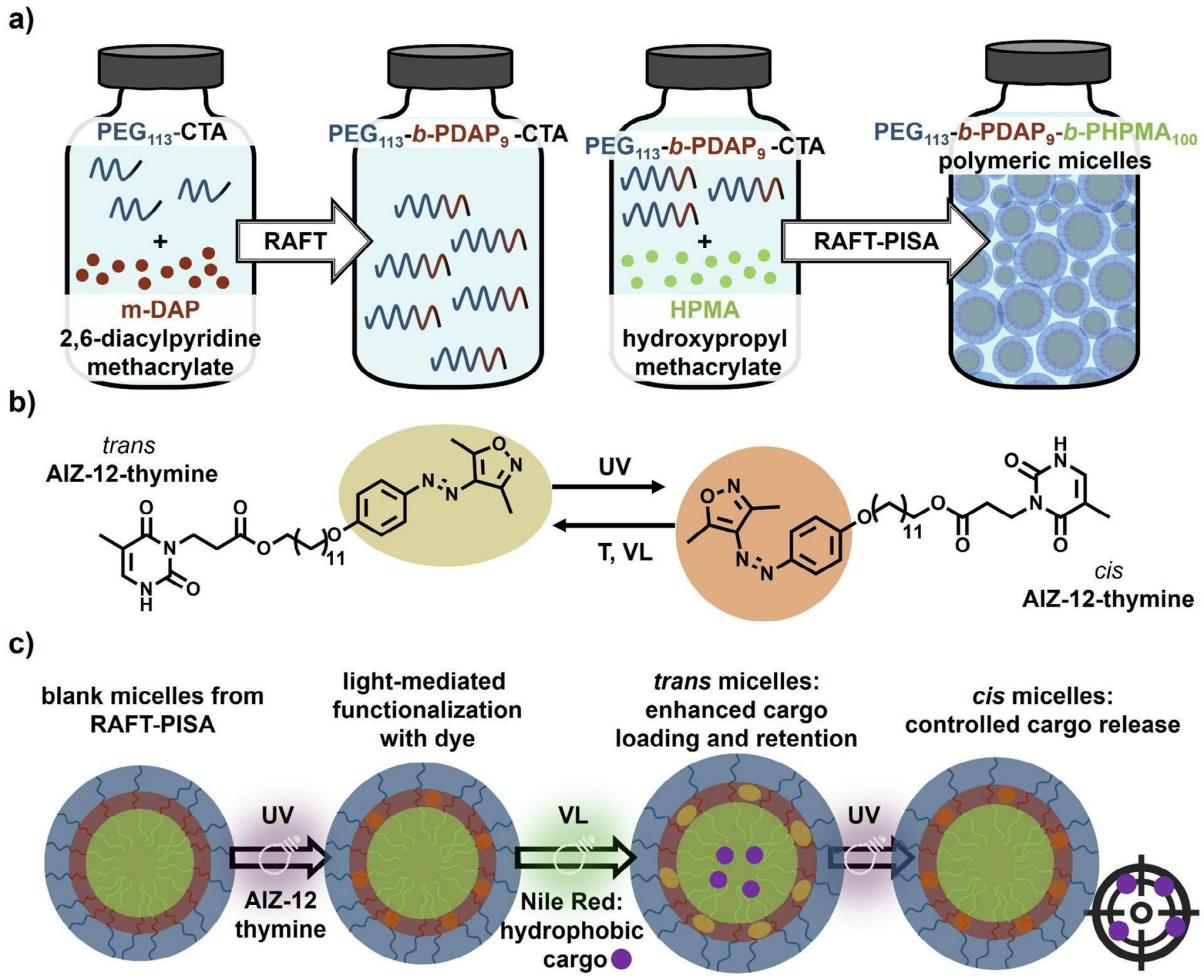
Overview of PISA‐assembled, AIZ‐doped micellar system for controlled release. a) Scheme of the elements used for the RAFT‐PISA self‐assembly into spherical micelles in water dispersion. First, an amphiphilic macro‐CTA is obtained by a RAFT polymerization between PEG_113_ (blue) and m‐DAP (red), mediated by a trithiocarbonate CTA (black). Then, micelles are obtained by RAFT‐PISA polymerization between the macro‐CTA and the HPMA (green) monomer. b) AIZ‐12‐thymine *trans* (yellow) and *cis* (orange) isomers. The *trans* to *cis* isomerization is triggered by UV irradiation, while the *cis* to *trans* isomerization can happen by visible light (VL) irradiation or by thermal relaxation (T). c) The micelles are functionalized by a light‐mediated protocol involving the UV‐induced isomerization of AIZ‐12‐thymine to its *cis* abundant state (orange). After that, the *trans* isomer (yellow) of the photoswitch is restored, and a model hydrophobic cargo (Nile Red, purple) is co‐encapsulated within the micelles core. Its release is enhanced after *trans‐*to*‐cis* photoisomerization of the AIZ‐12‐thymine anchored inside the micelles.

To address these interconnected limitations, in this study, we propose the supramolecular functionalization of micelles produced by RAFT‐PISA, as shown in**Figure** **1**a. The micelles used in this study consist of three polymer blocks: a poly(ethylene glycol) (PEG) outer shell forming for colloidal stability and biocompatibility, a poly(2,6‐diacylpyridine) (PDAP) midblock to enhance nanostructure stability, and a hydrophobic poly(2‐hydroxypropyl methacrylate) (PHPMA) core‐forming block that enables the PISA process.^[^ ^46^, ^47^ ^]^ An amphiphilic diblock copolymer (PEG_113_‐*b*‐PDAP_9_‐CTA) was first synthesized and used as a macro‐chain transfer agent (macro‐CTA) for the RAFT‐PISA of HPMA (2‐hydroxypropyl methacrylate) in water. The PDAP block, containing a nucleobase analog, allows for supramolecular functionalization via complementary nucleobase pairing after the synthesis of the micelles, through hydrogen bonding.^[^ ^46^, ^47^ ^]^ To exploit this, we synthesized a thymine‐functionalized arylazoisoxazole (AIZ‐12‐thymine, Figure 1b) and incorporated it into the aqueous micelles through a light‐mediated loading protocol, where UV‐induced *trans–cis* isomerization allowed the penetration of the dye, thus a stable functionalization of the micelles with the photoresponsive unit (Figure 1c). In this system, the hydrophobic *trans* isomer promotes the cargo loading and retention, whereas the more polar *cis* isomer enhances its release. Therefore, we have developed a new method for precisely modulating the loading and release of hydrophobic loads within water dispersions of micelles. This process is highly advantageous as it eliminates the need for organic solvents and maintains the stability of the micelle structure, even after multiple cycles of light irradiation. These features make our system a promising platform for controlled drug delivery.

## 2 Results and Discussion

### 2.1 Micelles Synthesis and Light‐Induced Functionalization

**Figure 2:**
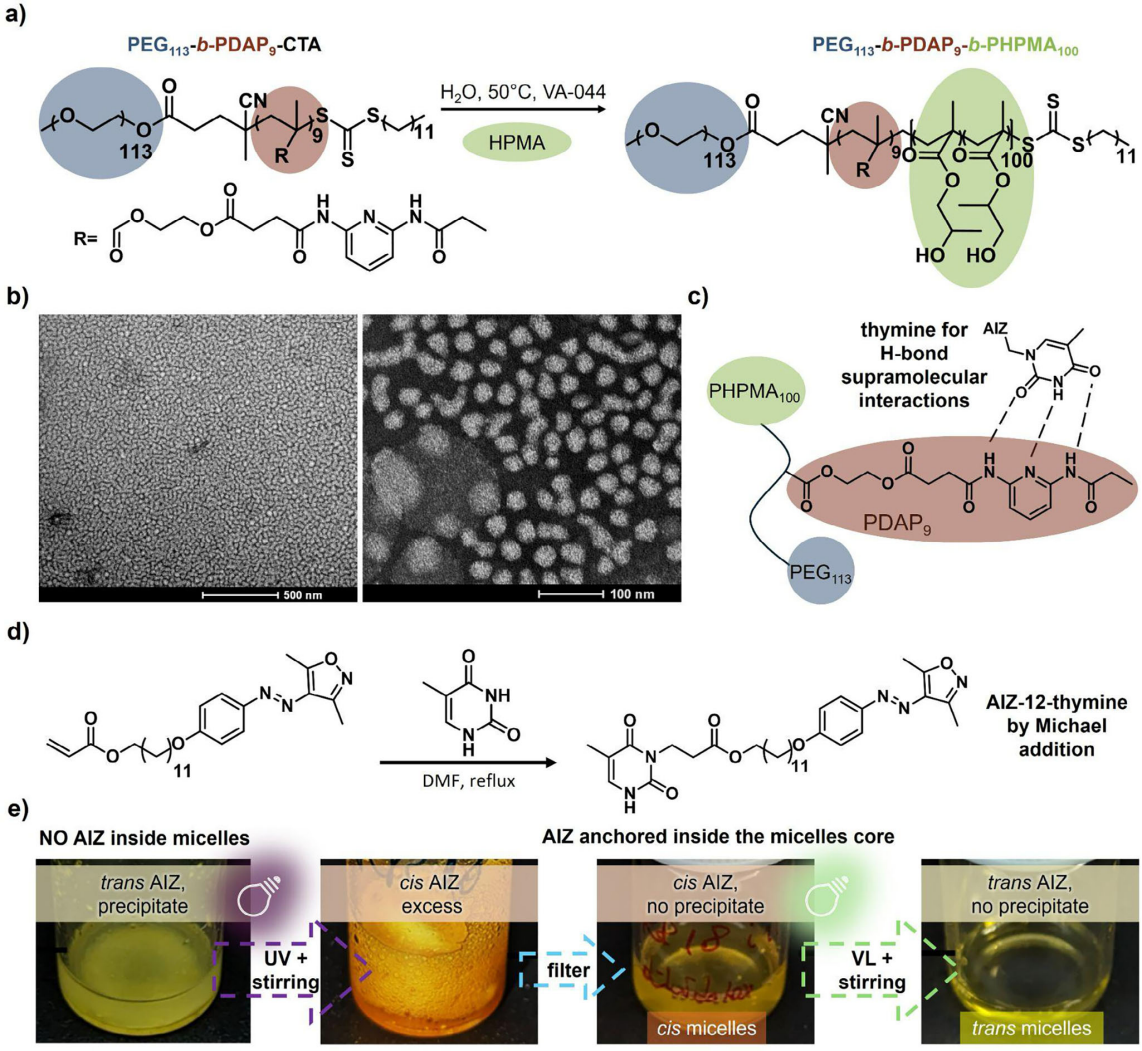
Synthesis and functionalization of the polymeric micelles. a) Scheme of the micelles synthesis by RAFT‐PISA polymerization. Reagents and conditions: HPMA, VA‐044, H_2_O, 50 °C, 2 h, quantitative conversion. b) TEM images of the synthesized micelles by RAFT‐PISA methodology. c) Scheme of molecular recognition for the supramolecular functionalization: the thymine group is linked to DAP units by hydrogen‐bonding in the micelles core. d) AIZ‐12‐thymine last synthetic step. Reagents and conditions: thymine, DMF, reflux, 18 h. e) Steps for the light‐mediated functionalization: mixing of *trans*‐AIZ‐12‐thymine with PISA micelles leads to photoswitch precipitation (hydrophobic exclusion). UV irradiation (365 nm, 6.3 mW cm^−2^, 2 h) generates *cis*‐AIZ‐12‐thymine, which diffuses into and stably anchors within the micelle core, yielding a colored emulsion after filtration (*cis* micelles). After VL irradiation (505 nm, 3.0 mW cm^−2^, 10 min) the *trans*‐AIZ‐12‐thymine is retained inside the micelles core, obtaining the *trans* micelles.

The RAFT polymerization reactions were carried out following previously reported procedures.^[^ ^46^ ^]^ Macro‐CTA (PEG_113_‐*b*‐PDAP_9_‐CTA) preparation details are provided in Experimental Section (Scheme S1 and Figure S1, Supporting Information). The RAFT‐PISA step (**Figure** **2**a) was performed by mixing HPMA with the macro‐CTA dispersed in water at 50 °C, using VA‐044 as a water‐soluble radical initiator. According to ^1^H‐NMR, complete monomer conversion was achieved within 2 hours (Figure S2, Supporting Information). Gel Permeation Chromatography (GPC) analysis of the final block copolymer PEG_113_‐*b*‐PDAP_9_‐*b*‐PHPMA_100_, confirmed controlled RAFT polymerization during the PISA process (Figures S3 and S4, Supporting Information). Successful micelles formation with a diameter of 33 ± 4 nm was confirmed by Transmission Electron Microscopy (TEM) images (Figure 2b) and Dynamic Light Scattering (DLS) that revealed a hydrodynamic diameter (*D_h_*) of 42 ± 1 nm and a low polydispersity index (PdI) of 0.1 (**Table** **1**).

**Table 1:** Size and dispersity of blank, *trans*‐, and *cis*‐functionalized micelles.

–	blank micelles	*trans* micelles	*cis* micelles
Diameter [nm]^a^ ^)^	33 ± 4	35 ±5	32 ± 4
*D_h_* [nm]^b)^	42 ± 1	46 ± 1	45 ± 2
PdI^c)^	0.1	0.1	0.1

^a)^ TEM core diameter ± SD;

^b)^ hydrodynamic diameter (*D_h_*) ± SD by DLS;

^c)^ polydispersity index (PdI).

To enable light‐responsiveness without incorporating the photoswitch directly into the polymer backbone, a thymine moiety was selected to anchor the DAP units in the micelle hydrophobic core via hydrogen bonding, as already well demonstrated by some of us,^[^ ^46^, ^47^ ^]^ (Figure 2c), introducing a photoresponsive switch without needing its direct incorporation into the polymer backbone.

In this case, we synthesized the AIZ‐12‐thymine, a novel arylazoisoxazole‐based photoswitch. Its final synthetic step involves a one‐pot Michael addition of a thymine derivative to an acrylate‐AIZ precursor, as shown in Figure 2d. This photoswitch combines the ability of AIZ to penetrate the micelles with the thymine‐mediated hydrogen bonding that ensures stable anchoring within the core. The detailed synthetic route is provided in Scheme S2 and Figures S6 and S7 (Supporting Information), while earlier steps are reported in the literature.^[^ ^48^ ^]^

The supramolecular functionalization of the micelles with the photoswitch AIZ‐12‐thymine was mediated by light, as it is schematized in Figure 2e. When the PISA dispersion (diluted to a concentration of 1 mg mL^−1^) is mixed with *trans*‐AIZ‐12‐thymine (either solid or dissolved in DMSO), the photoswitch is not able to enter into the micelle core and precipitates because of its hydrophobicity. In fact, by filtering the obtained mixture through a 0.45 µm pore size hydrophilic membrane, a colorless filtrate was obtained with no detectable photoswitch absorbance (Figure S8, Supporting Information), meaning the inclusion of the photoswitch inside the micelles did not take place. On the other hand, 1 min of irradiation of the unfiltered mixture at 365 nm (6.3 mW cm^−2^) converts AIZ‐12‐thymine into a *cis*‐rich photostationary state, allowing for the supramolecular functionalization. Although we cannot exclude a very low solubility of the *cis* isomer in water, we believe that its penetration through the PEG corona is mainly driven by its increased polarity and bent geometry. Under these conditions, filtration of the excess of photoswitch produces a stable, colored dispersion with no precipitate, assuming the successful integration of the photoswitch inside the micelles. This is further supported by the UV–vis spectra of the dispersion after filtration (Figure S9, Supporting Information), which confirm the presence of AIZ‐12‐thymine inside the micelles. The functionalized micelles containing the AIZ‐12‐thymine in its *cis‐*abundant state have been called *cis* micelles. When the same supramolecular strategy was attempted using a related thymine‐functionalized azobenzene, no incorporation into the micellar core was observed, even after UV irradiation (Figure S10, Supporting Information). Moreover, AIZ‐12‐thymine remains stably anchored within the micelles. Indeed, irradiation with visible light induces isomerization back to the *trans* form without causing precipitation, meaning the robust thymine–DAP hydrogen bonding ensures persistent photoswitch integration in both isomeric states, maintaining stability over extended storage periods. Indeed, after six months of storage, UV–vis and DLS analyses showed no spectral or dimensional changes (Figure S11, Supporting Information), confirming the long‐term stability of the AIZ‐12‐thymine anchoring and the absence of detectable photoswitch leakage. We refer to micelles containing predominantly the *trans* isomer of AIZ‐12‐thymine as *trans* micelles. Finally, to demonstrate how the thymine functionalization is crucial for the stable anchoring of the photoswitch, a different AIZ compound, AIZ‐12‐OH, showing the same structure of AIZ‐12‐thymine but lacking the thiamine moiety,^[^ ^48^ ^]^ was tested under the same light‐activated functionalization conditions (Figure S12, Supporting Information). This photoswitch can transiently penetrate (through hydrophobic partitioning) the micelles in its *cis*‐enriched state but precipitates within one day, confirming that hydrogen bonding between thymine and DAP units is essential for a stable incorporation.

### 2.2 Micelles Photoresponsive Behavior

**Figure 3:**
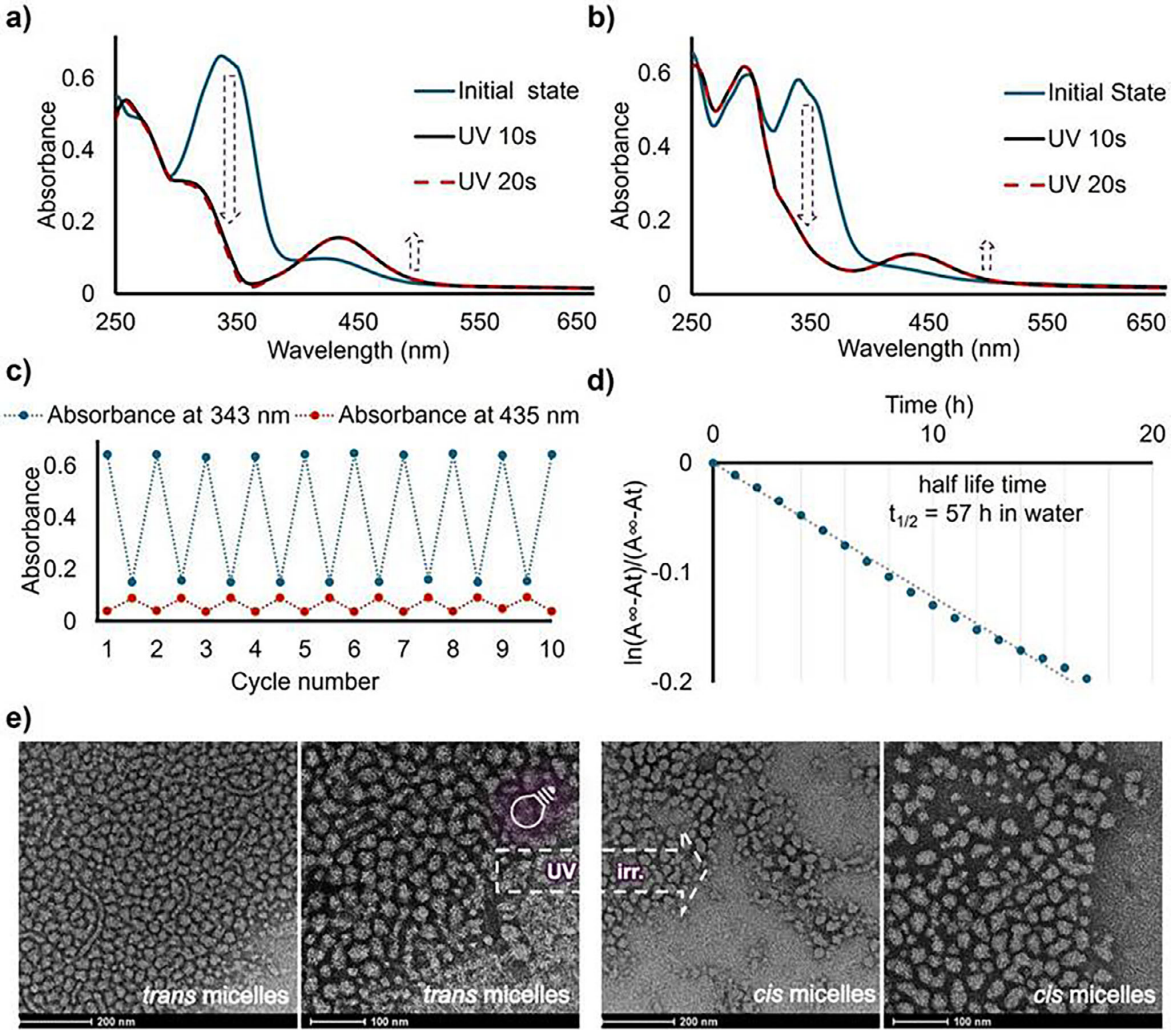
Spectroscopic characterization of AIZ‐12‐thymine and functionalized micelles. a) UV–vis absorption spectra of AIZ‐12‐thymine in methanol (0.1 mg mL^−1^, 1 mm cuvette) before and after UV irradiation (365 nm, 6.3 mW cm^−2^). b) UV–vis spectra of AIZ‐functionalized micelles in water (0.2 mg mL^−1^, 10 mm cuvette) before and after UV irradiation (365 nm, 6.3 mW cm^−2^). c) Evolution of the absorbance at 343 nm and 435 nm of AIZ‐12‐thymine in micelles dispersion (0.2 mg mL^−1^, 10 mm cuvette) after 10 cycles of UV light/visible light irradiation (visible light at 505 nm, 3.0 mW cm^−2^) to demonstrate a consistent *trans* to *cis* and *cis* to *trans* photoisomerization. d) Relaxation kinetics of *cis* micelles in water dispersion. e) TEM images of AIZ‐functionalized micelles before (*trans*‐micelles) and after (*cis‐*micelles) UV irradiation (365 nm, 6.3 mW cm^−2^).

The photoresponsive behavior of the AIZ‐12‐thymine was first studied by UV–vis spectroscopy, both in methanol solution and inside the micelle dispersion. In methanol, AIZ‐12‐thymine shows a π→π* band at 338 nm and an n→π* band at 442 nm (**Figure** **3**a), typical of azo compounds. Upon irradiation at 365 nm (6.3 mW cm^−2^), the spectra recorded after 10 and 20 s of irradiation overlap, indicating that the *cis*‐rich photostationary state is reached within the first 10 s. In this state, the π→π* band intensity decreases markedly while the n→π* band grows. The UV–vis spectra of AIZ‐12‐thymine functionalized micelles dispersed in water are shown in Figure 3b. In the *trans*‐micelles, both π→π* and n→π* bands shift slightly to longer wavelengths (343 and 435 nm, respectively) if compared to methanol. UV irradiation of the *trans*‐micelles reproduces the fast isomerization observed in the methanol solution: a *cis*‐rich photostationary state forms in less than 10 s, while the DAP absorption ≈290 nm remains unchanged. To quantify the photoswitch incorporation in the micelles, the dye‐doped micelles were lyophilized to break up the assemblies, then redispersed in methanol and estimated using the molar absorption coefficient at 338 nm ε = 21 902 m ^−1^ cm^−1^ (Figure S13, Supporting Information). A concentration of ≈8.6 × 10^−2^ mg mL^−1^, corresponding to 8.6 wt% relative to the micelles' weight, was determined. Since unincorporated photoswitch had precipitated before filtration, the micelles dispersion can be considered as oversaturated. This measured concentration, therefore, represents the maximum achievable loading under our experimental conditions. Moreover, we demonstrated the stability and fatigue resistance of the functionalized micelles by subjecting them to multiple cycles of UV and visible light irradiation in water. No decrease in absorbance was observed after repeated cycling (Figure 3c), confirming the robustness of the system. Therefore, the switching between *trans* and *cis* micelles can be dynamically and reversibly modulated using light alone. Since the *cis* micelles thermally relax back to the *trans* state, we monitored the changes in the absorption spectra of the micelles in the dark after reaching the photostationary state. From these measurements, we determined the half‐life of the *cis* isomer in the micelles. By fitting the decay of the maximum of the n→π* absorption band with a first‐order kinetic model, we extracted a rate constant *k* = 0.0122 h^−1^, corresponding to a half‐life (t_1_/_2_) of ≈57 h (Figure 3d). This markedly exceeds the ≈32 h *cis*‐isomer half‐life measured in methanol (Figure S14, Supporting Information), indicating that the hydrophobic micelle core significantly stabilizes the *cis* form. Such an extended lifetime is particularly advantageous for applications requiring sustained micelle modulation without repeated illumination pulses.

After UV‐induced conversion from *trans* to *cis‐*micelles, TEM images (Figure 3e) show the appearance of minor surface irregularities, as overall integrity and spherical shape are both nicely preserved. Additionally, AIZ‐12‐thymine incorporation does not significantly alter micelle morphology (the comparison between blank micelles and *trans‐*micelles is reported in Figure S15, Supporting Information). Image analysis gives average diameters of 33 ± 4 nm (blank), 35 ± 5 nm (*trans*), and 32 ± 4 nm (*cis*) (Table 1). Statistical analysis (unpaired *t*‐test) revealed significant differences between all three micellar populations. The corresponding *p‐*values are reported in Table S1 (Supporting Information). As *p* values < 0.05 demonstrate reproducible differences among the three populations, the absolute shift in the micelles diameter is small. The expansion in the *trans‐*micelles reflects the bulkier *trans* photoswitch in the core, while shrinkage in the *cis‐*micelles is consistent with the more compact *cis* form. DLS further confirms these trends: the *D_h_* grows by ≈8% upon AIZ loading (from 42 ± 1 to 46 nm ± 1), but photoisomerization does not significantly change *D_h_* (Figure S16, Supporting Information). This indicates that although the core responds to light, the overall hydrodynamic envelope remains stable.

### 2.3 Light‐Controlled Loading and Release of Nile Red in Functionalized Micelles

**Figure 4:**
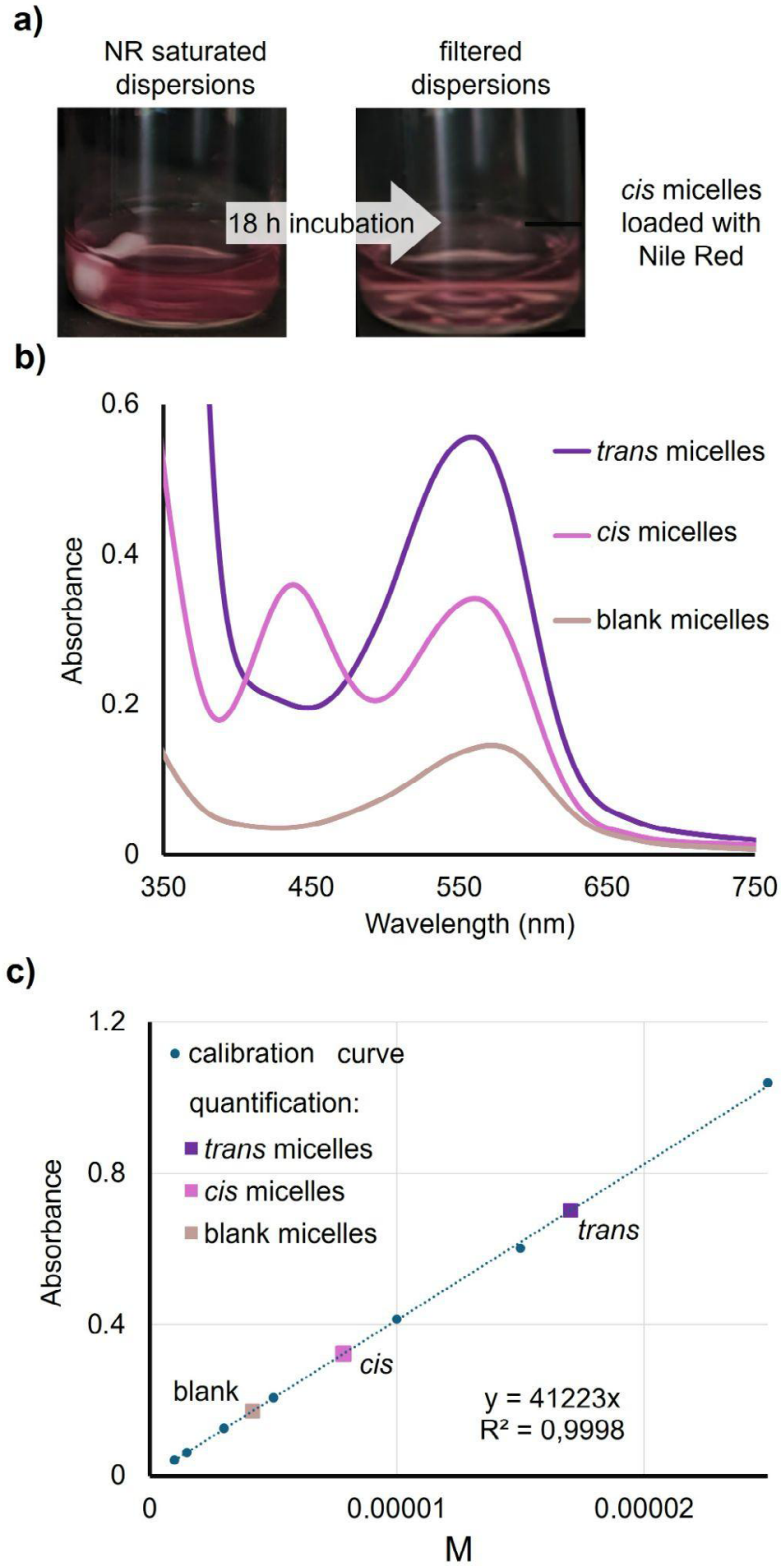
Nile Red encapsulation in blank and AIZ‐functionalized micelles. a) Nile Red loading scheme: incubation of Nile Red with micelles (*cis* micelles taken as example) and removal of unencapsulated dye by 0.45 µm pore filtration. b) UV–vis spectra of the filtrates from blank, *cis*‐micelles, and *trans*‐micelles, once lyophilized and solved in methanol. c) Calibration curve for Nile Red at 550 nm and quantified encapsulation levels.

Based on the collected photoresponse data, we evaluated how AIZ‐12‐thymine affects the loading and release of Nile Red, a hydrophobic fluorescent dye with a strong absorption band near 550 nm (Figure S17, Supporting Information, spectrum in DMSO) widely used as a probe to mimic a hydrophobic drug. The micelle solutions were directly added to Nile Red powder, as described in Experimental Section. Blank micelles, *trans*‐micelles and *cis*‐micelles (the latter obtained by 1 min UV irradiation at 365 nm, 6.3 mW cm^−2^, then stored in the dark) with a concentration of 1 mg mL^−1^ were each added to the Nile Red powder and stirred for 18 h. Unencapsulated dye was removed by filtration through a 0.45 µm pore size cellulose‐acetate membrane (**Figure** **4**a), yielding clear, colored dispersions. UV–vis spectra of the filtered samples (Figure 4b) reveal that *trans*‐micelles exhibit the highest absorbance in the Nile Red region (550–560 nm), along with the characteristic AIZ π→π* and n→π* bands. In *cis*‐micelles, more than 80% of the AIZ‐12‐thymine remains in the *cis* isomer after 18 hours of loading (derived from the relaxation kinetics of AIZ‐micelles). These micelles show a lower Nile Red absorbance signal, which partially overlaps with the AIZ n→π band, while blank micelles display the weakest signal overall. A progressive blue shift from blank to *cis*‐ to *trans*‐micelles indicates a decrease in the polarity of the micellar core and stronger Nile Red–core interactions in the *trans* state. For completeness, the unedited, full‐range UV–vis spectra corresponding to Figure 4b are provided in Figure S18 (Supporting Information). Quantification was carried out by lyophilizing each sample, redissolving the resulting solids in methanol, and comparing the absorbance with a calibration curve (Figure 4c). Concentrations of encapsulated Nile Red are 4.2 × 10^−6^ ± 0.4 × 10^−6^ m for blank micelles, 7.8 × 10^−6^ ± 0.2 × 10^−6^ m for *cis*‐micelles, and 1.7 × 10^−5^ ± 0.1 × 10^−5^ m for *trans*‐micelles, corresponding to 0.13, 0.25, and 0.54 wt%, respectively. These results demonstrate the AIZ functionalization to significantly enhance the Nile Red loading, with the low‐polarity *trans* core favoring the highest encapsulation efficiency.

**Figure 5:**
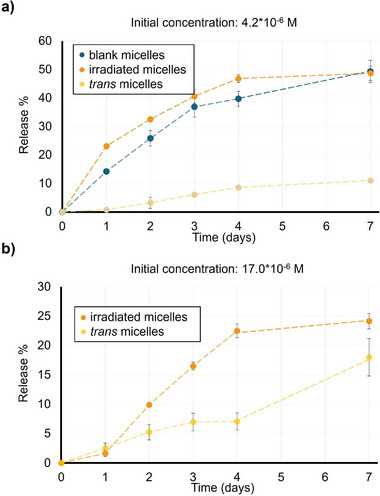
Effect of light on the Nile Red releases kinetics. a) Release profiles of Nile Red from blank, *trans‐*functionalized and irradiated micelles at initial loadings of 4.2 × 10^−6^ m. b) Release profiles of Nile Red from *trans‐*AIZ and irradiated micelles at initial loadings of 17.0 × 10^−6^ m.

Indeed, the presence of the photoswitch is expected to also greatly affect the release from the micelles. To evaluate light‐controlled release, we employed a dialysis setup detailed in Figure S19 (Supporting Information). Blank and AIZ‐doped micelles loaded with Nile Red were placed inside a dialysis device with a 3.5 kDa MWCO membrane, immersed in Milli‐Q water. The Nile Red loading of the micelles is detailed in Experimental Section, as two different initial concentrations of 4.2 × 10^−6^ m and 17.0 × 10^−6^ m were used. To assess the effect of the AIZ‐12‐thymine isomeric state on Nile Red release, *trans*‐micelles doped with Nile Red were divided in two aliquots: one maintained in the *trans* state, the other irradiated at 365 nm (6.3 mW cm^−2^) to enrich *cis* content right before the start of the release experiment, after verifying that UV light exposure under the applied conditions does not induce photobleaching of Nile Red (Figure S20, Supporting Information). Blank micelles served as a control. For the initial concentration of 4.2 × 10^−6^ m, irradiated micelles had released ≈23% of the dye after 1 day, blank micelles ≈14%, and *trans*‐state micelles less than 1% (**Figure** **5**a). By day 7, both blank and irradiated micelles converged on ≈49% release, while *trans*‐micelles released ≈11% of their initial Nile Red load. These results demonstrate that UV‐triggered *trans*‐*cis* isomerization accelerates Nile Red diffusion, as *cis*‐rich micelles release their cargo at rates similar to unfunctionalized micelles, whereas *trans*‐rich cores effectively retain the dye.

However, the addition of the photoswitch system also allows for the incorporation of higher amounts of dye; therefore, the same experiments were performed on micelles bearing the highest possible loading of Nile Red (17.0 × 10^−6^ m; Figure 5b). After splitting into *trans* and irradiated aliquots and tracking release (Figure 5b), irradiated samples released ≈23% of their payload by day 4 compared to just ≈6% from *trans* micelles. This again confirms that *cis*‐state enhances release, while the *trans* state prioritizes the retention of the payload. The comparison between both loading regimes reveals a trade‐off between fractional release and total cargo delivered. At 4.2 × 10^−6^ m, *cis*‐micelles released ≈50% in seven days; at 17.0 × 10^−6^ m, only ≈23% was released in the same period. However, 23% of the higher load corresponds to a larger absolute amount of Nile Red than 50% of the lower load. Thus, by adjusting the initial payload, one can tune the balance between sustained fractional release and overall dose delivered, adding an additional design parameter for programmable, light‐activated therapeutic regimens. A complementary experiment, illuminating the photoresponsive micelles every 24 h, confirmed that repeated light exposure further accelerates Nile Red release (Figure S21, Supporting Information). Under these conditions, cumulative release reached 28% and 71% after 4 days when the initial concentrations of Nile Red were 17 × 10^−6^ m and 4.2 × 10^−6^ m, respectively, highlighting the reproducibility and tunability of the photoinduced release process.

## 3 Conclusion

In this study, we developed a light‐responsive micellar system by incorporating a thymine‐functionalized arylazoisoxazole photoswitch into RAFT‐PISA–assembled nanoparticles. The unique design of AIZ‐12‐thymine enables the micelles to be functionalized only when light irradiation is used, since only the polar *cis* isomer can penetrate the PEG corona and diffuse into the hydrophobic core. There, thymine–DAP interactions lock the photoswitch in place, so that even after isomerization to the apolar *trans* form, the dye remains stably integrated without the need for organic co‐solvents for very long times (more than 6 months).

Under these purely aqueous conditions, we achieved high loadings for photoresponsive micelles (8.6 wt% AIZ‐12‐thymine and 0.54 wt% Nile Red) without compromising the particle integrity. Transmission electron microscopy (TEM) and dynamic light scattering (DLS) confirm that neither functionalization nor repeated *trans–cis* cycling disrupts micelle size or dispersity, demonstrating robust morphological stability. A 365 nm irradiation converts the photoswitch to its *cis* form, increasing core polarity and triggering accelerated Nile Red release; the long thermal half‐life of the *cis* state (≈57 h) maintains the activated release profile over several days without further irradiation. Furthermore, we show that initial Nile Red loading provides an additional tuning parameter, higher loadings, and slow release kinetics, offering a precise control over delivery kinetics.

Taken together, our results establish a scalable platform that marries PISA morphological precision with the durable, aqueous‐compatible photoswitching of AIZ‐12‐thymine. This strategy paves the way for on‐demand, spatiotemporally controlled drug delivery, and future work will explore red‐shifted AIZ variants as well as in vitro and in vivo evaluations toward clinical translation.

## 4 Experimental Section

### Materials

Poly(ethylene glycol) monomethyl ether (PEG_113_‐OH), with a molecular weight of 5000 g mol^−1^ and the chain transfer agent 4‐cyano‐4‐[(dodecylsulfanylthiocarbonyl)sulfanyl]pentanoic acid (97%) (CTA) were obtained from Merck and used without further purification. 2,2′‐Azobis(2‐methylpropionitrile) (AIBN) was purchased from Merck and recrystallized in ethanol before use. Dioxane, also acquired from Merck, was purified by passing it through a basic alumina column. 2,2′‐Azobis[2‐(2‐imidazolin‐2‐yl)propane] dihydrochloride (VA‐044) was obtained from TCI (TCI Europe N.V., Zwijndrecht, Belgium). The monomer 2‐hydroxypropyl methacrylate (97%, a mixture of isomers 2‐hydroxypropyl methacrylate and 1‐hydroxypropan‐2‐yl methacrylate, HPMA) was purchased from Merck and purified by alumina column filtration to remove the inhibitor. The methacrylate monomer m‐DAP, PEG_113_‐*b*‐PDAP_9_‐CTA macro‐CTA and PEG_113_‐*b*‐PDAP_9_‐*b*‐HPMA_100_ micelles were synthesized according to previously reported procedures.^[^ ^46^, ^47^ ^]^ The synthesis of AIZ‐12‐thymine involved the preparation of the acrylate precursor 12‐(4‐((3,5‐dimethylisoxazol‐4‐yl)diazenyl)phenoxy)dodecyl acrylate, following previously reported methods.^[^ ^48^ ^]^

### Synthesis and Characterization

For a detailed description of the materials synthesis and characterization, please see Supporting Information. ^1^H‐NMR spectra were acquired on a Bruker AV 400 spectrometer (Bruker, Billerica, MA, USA) operating at 400 MHz proton frequency at 25 °C with standard pulse sequences. Chemical shifts are reported in ppm relative to TMS as the internal standard (δ = 0.00 ppm). The ^13^C‐NMR spectra were acquired on a Varian Inova 400 MHz; the notations s, d, t, m, Ar indicate respectively: singlet, doublet, triplet, multiplet, aromatics.

### GPC Measures

GPC was performed using a Waters Alliance 2695 liquid chromatography system equipped with a UV‐vis‐Waters 2998 Photodiode Array detector. Two Waters Styragel columns (HR1, 100 Å, and HR3, 1000 Å) were employed (5 µm, 7.8 × 300 mm) with HPLC‐grade DMF containing 50 mm LiBr as the eluent at a flow rate of 0.5 mL min^−1^. Samples for GPC were prepared by taking an aliquot of the polymer dispersion, lyophilizing it, and dissolving it in DMF (50 mm LiBr), obtaining a sample concentration of 1 mg mL^−1^.

### DLS Measures

DLS measurements were performed using a Malvern Instrument Nano Zs (Malvern, Worcestershire, UK) equipped with a He─Ne laser at 633 nm and a detector angle of 173° at 25 °C. The self‐assembled aqueous dispersions were measured at an approximate concentration of 1.0 mg mL^−1^, and the hydrodynamic diameters (*D*_h_) were reported as the average of three measurements per sample to ensure reproducibility.

### TEM Measures and Statistical Analysis

TEM was conducted on a FEI Tecnai T20 microscope (FEI Company, Waltham, MA, USA) operating at 200 kV. TEM samples were prepared by placing 10 µL of each self‐assembly dispersion (≈1.0 mg mL^−1^) onto continuous carbon film‐copper grids. Excess liquid was removed by capillary action using filter paper. The grids were then stained with 1% aqueous uranyl acetate, with the excess stain removed in the same manner. Grids were dried overnight under vacuum. Micelle diameters (in nm) were measured from TEM images using *ImageJ* software. For each population, the diameters of 100 individual micelles (*n* = 100) were analyzed. Statistical analysis (unpaired *t*‐test) revealed significant differences between all three micelle populations, with *p* values reported in Table S1 (Supporting Information).

### Micelles Filtration

To remove solid residues from micelles saturated with hydrophobic compounds, cellulose acetate syringe filters with a 0.45 µm pore size (Millipore) were employed.

### UV–Vis Measures and *cis* Isomer Half‐Life Time Measures

The UV–visible spectra were recorded on a UV–vis Varian Cary 400 spectrometer at room temperature. The UV irradiations were carried out with a M365L3‐C1 (Thor‐Labs) lamp with an irradiation power of 6.3 mW cm^−2^ calculated at a distance of 3 cm from the lamp and a wavelength of 365 nm. The visible light irradiations were carried out with a M505L4 (Thor‐Labs) lamp with an irradiation power of 3.0 mW cm^−2^ calculated at a distance of 3 cm from the lamp and a wavelength of 505 nm. The half‐life of the *cis* isomer was calculated by plotting the change in absorbance at 353 nm over time. Assuming a first‐order kinetics process, characteristic half‐life times (t_1/2_) for all compounds were calculated, using Equations (1) and (2):

First order rate constant K

(1) $$\ln\left(A_{\infty }-A_{t}\right)/\left(A_{\infty }-A_{0}\right)=-\mathrm{Kt}$$

where A_∞_ represents the absorption intensity of AIZ after full *cis*‐to‐*trans* isomerization, A_t_ signifies the absorption intensity of AIZ maintained in darkness for a duration “t,” and A_0_ represents the absorption intensity of azobenzenes at the photostationary state following UV light irradiation.

Thermal isomerization half‐life t_1/2_

(2) $$t_{1/2}=\ln\left(2\right)/K$$

where k is the first‐order kinetics constant calculated by Equation 1.

### Nile Red Loading Procedures

Nile Red loading into the different micelles was performed using two distinct methods, depending on the required concentration. Regarding the 4.2 × 10^−6^ m Nile Red concentration, we first prepared a thin film of Nile Red by evaporating a concentrated dichloromethane solution under reduced pressure. The dried film was then rehydrated directly with the micelle dispersion and magnetically stirred for 18 h. This procedure ensured quantitative and precise loading of Nile Red into the micelle cores. For the blank micelles, the Nile Red was added directly as a powder in a quantity exceeding the loading capacity of the micelles; thus, after the encapsulation time of 18 h, the dispersion was filtered. The same was done for the AIZ micelles with a Nile Red concentration of 17.0 × 10^−6^ m.

### Dialysis Experiments

The Nile Red release was monitored by dialysis using a Slide‐A‐Lyzer MINI Dialysis Device (3.5 kDa MWCO, 0.5 mL; Thermo Fisher Scientific) immersed in Milli‐Q water (14 mL) at room temperature under magnetic stirring (200 rpm). At specified time intervals, the sample was extracted from the dialysis cup, its UV–vis spectrum was recorded, and it was then returned to the device for continued dialysis.

## Conflict of Interest

The authors declare no conflict of interest.

## Supporting information



Supporting Information

## Data Availability

The data that supports the findings of this study are available in the supplementary material of this article.
