# Supplementary material for: Light‐Mediated Supramolecular Functionalization of Polymerization‐Induced Self‐Assembled Micelles
Source: Small. 2025 Nov 6;21(51):e11176. doi: 10.1002/smll.202511176 (PMC12723323; doi:10.1002/smll.202511176)
Supplement: Supplementary file 1 — Supporting Information [file SMLL-21-e11176-s001.docx]

**Supporting Information**

**Light-mediated Supramolecular Functionalization of Polymerization-Induced Self-Assembled Micelles**

Ruggero Rossi, Miriam Abad, Luis Oriol, Daniele Martella, Camilla Parmeggiani*, Milagros Piñol*

**Supramolecular Functionalization of micelles as Photoresponsive Nanocarriers
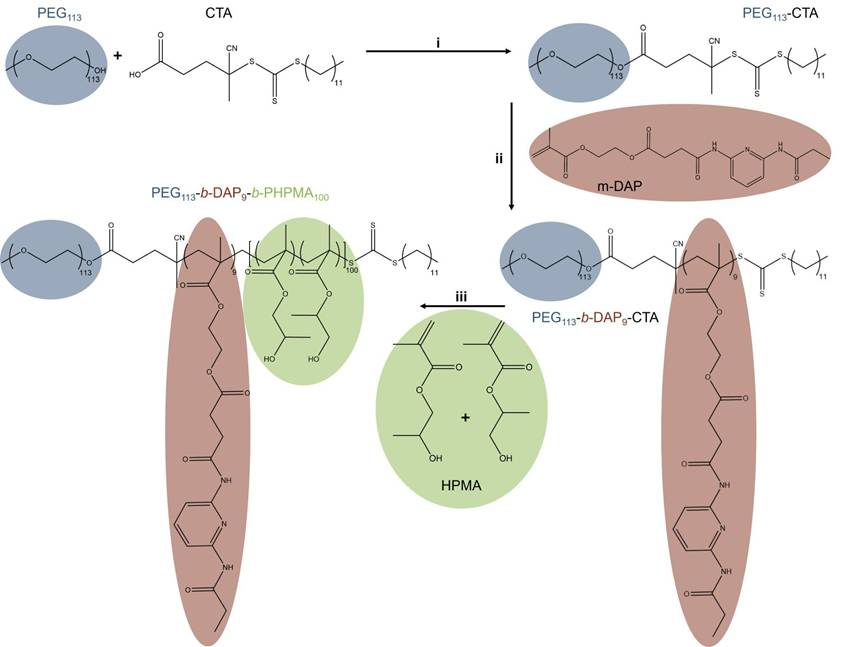
**

**Scheme S1: macro-CTAs and micelles synthesis scheme:** i) PEG_113_-CTA synthesis (Steglich esterification): 4-cyano-4-[(dodecylsulfanylthiocarbonyl)sulfanyl]pentanoic acid, PEG_113_, DMAP, DCC, dichloromethane, r.t., 21 h, yield = 81 %; ii**)** PEG_113_-*b*-PDAP_9_-CTA synthesis (RAFT polymerization)**:** PEG_113_-CTA, m-DAP, AIBN, dioxane, 75 °C, 5 h, yield = 72 %; iii) PEG_113_-*b*-PDAP_9_-*b*-HPMA_100_ micelles synthesis (RAFT-PISA polymerization): PEG_113_-*b*-PDAP_9_-*b*-HPMA_100_, VA-004, H_2_O, 50 °C, 2 h, quantitative.

**Poly(ethylene glycol) methyl ether 4-cyano-4-[(dodecylsulfanylthiocarbonyl)sulfanyl] pentanoate (PEG_113_-CTA) synthesis:**

Poly(ethylene glycol) monomethyl ether (PEG_113_-OH, molar mass approx. 5000) (1.48 g, 0.3 mmol), 4-cyano-4-[(dodecylsulfanylthiocarbonyl)sulfanyl]pentanoic acid (0.35 g, 0.87 mmol), and 4-(dimethylamino)pyridine (0.03 g, 0.24 mmol) were dissolved in 15 mL of dry dichloromethane under argon atmosphere. The mixture was cooled to 0 °C, after which *N,N′*-dicyclohexylcarbodiimide (0.27 g, 1.3 mmol) was added. The solution was stirred for 1 h at 0 °C and then for 21 h at room temperature. The resulting white precipitate was filtered off, and the solvent was evaporated under reduced pressure. The crude product was precipitated twice in cold diethyl ether, centrifuged, and dried, obtaining 1.0 g of PEG_113_-CTA as a yellow powder with an 81 % yield.

**PEG_113_-*b*-PDAP_9_-CTA synthesis:**

PEG_113_-CTA (0.60 g, 0.11 mmol), monomer m-DAP (0.42 g, 1.1 mmol), and AIBN (2.72 mg, 0.017 mmol) were added to a crimp vial and dissolved in 2.5 mL of dry dioxane. The solution was purged with argon for 20 min and then placed in a preheated oil bath at 75 °C for 5 h. Afterward, the reaction mixture was cooled in an ice bath and exposed to air. The crude product was precipitated twice in cold diethyl ether, then centrifuged and dried under vacuum overnight. 0.86 g of PEG_113_-*b*-PDAP_9_-CTA were obtained as a pale yellow powder with an 84% yield. From ^1^H-NMR was estimated a 92% conversion of m-DAP.

In order to estimate the degree of polymerization of the PDAP block, the relative integration of proton signal at 3.37 ppm, corresponding to PEG block methyl terminal group was compared to the average calculated on signals at 4.41-4.08 ppm and 2.89-2-60 ppm, corresponding to DAP repeating unit, obtaining a 9.2 ratio between those normalized signals (degree of polymerization approx. 9).

^1^H-NMR (Figure S1, 400 MHz, CDCl_3_) δ: 8.97-8.23 (m, **PDAP**, -N**H**, 18H), 7.94-7.52 (m, **PDAP**, Ar, 25H), 4.41-4.08 (m, **PDAP**, O-C**H_2_**-C**H_2_**-O, 38H), 3.83-3.43 (m, **PEG**, O-C**H_2_**-C**H_2_**-O), 3.37 (s, **PEG**, O-C**H_3_**, 3H), 2.89-2.60 (m, **PDAP**, O=C-C**H_2_**-C**H_2_**-C=O, 36H), 2.44-2.31 (m, **PDAP**, O=C-C**H_2_,** 18H), 1.34-0.78 (m,**PDAP**, -C**H_2_**- + -CH_2_-C**H_3_**, 72H) ppm.

**PEG_113_-*b*-PDAP_9_-*b-*PHPMA_100_ PISA micelles synthesis:**

PEG_113_-*b*-PDAP_9_-CTA (0.075 g, 8.0 μmol), HPMA (0.12 g, 0.83 mmol), and VA-044 (0.8 mg, 2.5 μmol) were added to a 10 mL crimp vial and dissolved in 0.78 mL of distilled water. The mixture was degassed by bubbling argon for 15 min, then placed in an oil bath at 50 °C. After 2 h, the vial was exposed to air and cooled to room temperature. Conversion: >99 %.

^1^H-NMR (Figure S2, 400 MHz, MeOD) δ: 7.81-7.58 (m, **PDAP**, Ar-**H**), 4.23-4.12 (m, **PDAP**, O-C**H_2_**-C**H_2_**-O), 4.08-3.94 (m, **PHPMA**,-C**H**-OH), 3.94-3.76 (m, **PHPMA**, O-C**H_2_**-C), 3.76-3.51 (m, **PHPMA**, O-C**H_2_**-C + **PEG**, O-C**H_2_**-C**H_2_**-O), 2.82-2.71 (m, **PDAP**, O=C-C**H_2_**-C**H_2_**-C=O), 2.49-2.35 (m, **PDAP**, O=C-C**H_2_**), 2.12-1.83 (m, **PHPMA**, -C**H_2_**-C-), 1.37-0.85 (m, **PHPMA**, C-C**H_3_** + **PDAP**, -C**H_2_**- + -CH_2_-C**H_3_**).


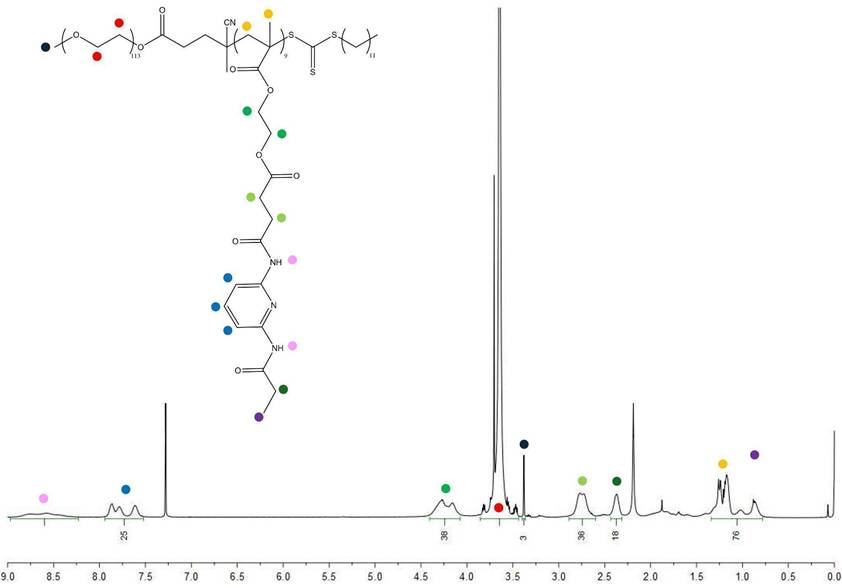


**Figure S1:** ^1^H-NMR (400 MHz, CDCl_3_) of **PEG_113_-*b*-PDAP_9_-CTA**.


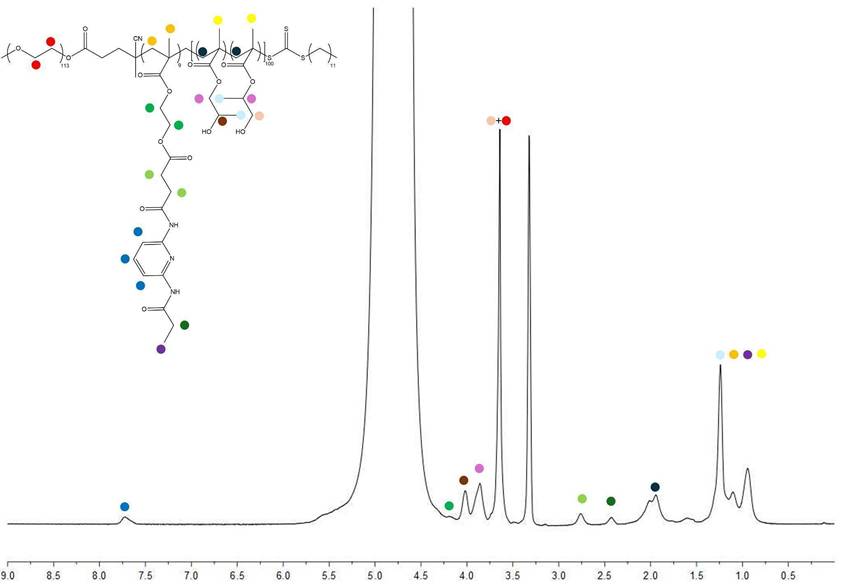


**Figure S2:** ^1^H-NMR (400 MHz, MeOD) of **PEG_113_-*b*-PDAP_9_-*b*-PHPMA_100_** (PISA micelles in water), after 2 h of reaction. The absence of methacrylate signals from HPMA means a quantitative conversion.


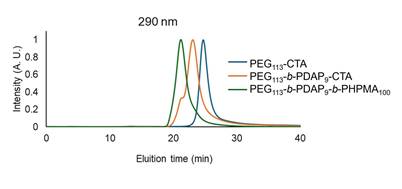


**Figure S3:** analysis by GPC of the **PEG_113_-*b*-PDAP9-*b*-PHPMA_100_** and comparison with the chromatograms of the macro-CTAs at 290 nm, showing a systematic shift to shorter elution times as the block copolymer molecular weight increased, confirming successful chain extension from the macro-CTA to the micelle-forming polymer.


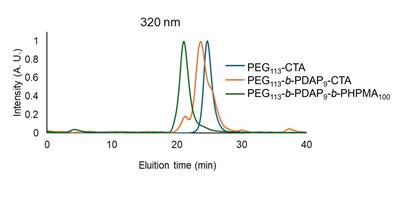


**Figure S4:** analysis by GPC of the **PEG_113_-*b*-PDAP9-*b*-PHPMA_100_** and comparison with the chromatograms of the macro-CTAs at 320 nm, showing a systematic shift to shorter elution times as the block copolymer molecular weight increased, confirming successful chain extension from the macro-CTA to the micelle-forming polymer.


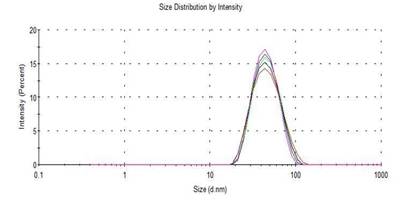


**Figure S5:** DLS measurements on pristine micelles (repeated 5 times on the same sample), resulting in a *D_h_* of 42 ± 1 nm, with a PdI of 0.1.


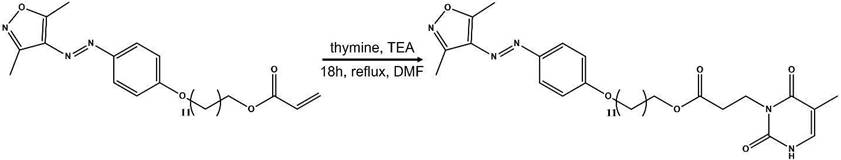


**Scheme S2: AIZ-12-thymine** (Michael addition): arylazoisoxazole acrylate (reagent), thymine, TEA, DMF, reflux, 18h, yield: 75%.

**AIZ-12-thymine synthesis**

12-(4-((3,5-dimethylisoxazol-4-yl)diazenyl)phenoxy)dodecyl acrylate (100.0 mg, 0.219 mmol) was dissolved in *N,N’*-dimethylformamide (2 mL) with thymine (83 mg, 0.66 mmol), and triethylamine (TEA) (44.3 mg, 0.44 mmol). The mixture was stirred for 18 h at reflux temperature then it was washed with water and ethyl acetate. The organic layers were dried over sodium sulphate and the solvent was removed under reduced pressure. The product was purified by elution on a silica gel column with diethyl ether and ethyl acetate (4:1) to obtain the pure product as a yellow solid (98.0 mg, 0.16 mmol, 75% yield).

^1^H-NMR (Figure S6, CDCl_3_, 400 MHz): δ = 7.87 – 7.68 (m, 2H, isoxazole-**H**), 7.19 (s, 1H, Ar-**H**), 7.04 – 6.90 (m, 2H, isoxazole-**H**), 4.09 (t, J = 6.8 Hz, 2H, CH_2_-C**H_2_**-N-), 4.03 (t, J = 6.6 Hz, 2H, O-C**H_2_**- CH_2_-), 3.96 (t, J = 6.0 Hz, 2H, CH_2_-C**H_2_**-O-), 2.76 (t, J = 6.1 Hz, 2H, -C=O-C**H_2_**- CH_2_-), 2.72 (s, 3H, isoxazole-C**H_3_**), 2.52 (s, 3H, isoxazole-C**H_3_**), 1.91 (s, 3H, thymine-C**H_3_**), 1.85 – 1.77 (m, 2H, alyphatic -C**H_2_**-), 1.62 – 1.56 (m, 2H, alyphatic -C**H_2_**-), 1.51-1.22 (m, 16H, alyphatic -C**H_2_**-) ppm.

^13^C-NMR (Figure S7, CDCl_3_, 100 MHz): δ 171.47 (s, **C**=O), 168.02 (s, isoxazole), 164.31 (s, **C**=O), 161.40 (s, Ar), 153.89 (s, isoxazole), 150.83 (s, **C**=O), 147.06 (s, Ar), 141.60 (d, NH_2_-**C**H=C), 132.19 (s, isoxazole), 123.89 (d, 2C, Ar), 114.62 (d, 2C, Ar), 110.48 (s, C-**C**H=CH_2_), 68.33 (t, O-**C**H_2_-), 65.28 (t, -**C**H_2_-O-), 45.01 (t, N-**C**H_2_-C), 33.07 (t, CH_2_-**C**H_2_-C=O), 29.06 (m, 8C, -**C**H_2_- aliphatic), 28.49 (t, -**C**H_2_- aliphatic), 25.90 (t, -**C**H_2_- aliphatic), 12.25 (q, **C**H_3_), 12.04, (q, **C**H_3_), 11.61 (q, **C**H_3_) ppm.


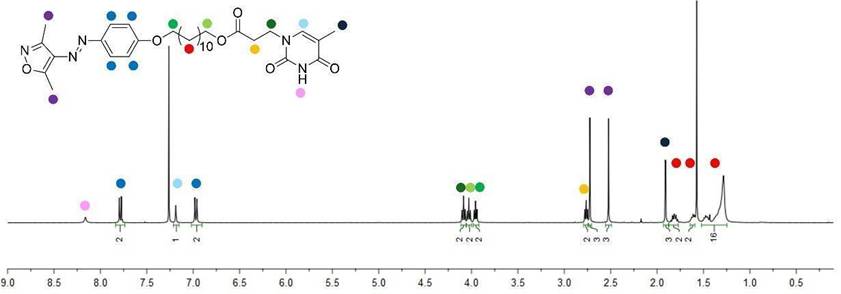


**Figure S6:** ^1^H-NMR (400 MHz, CDCl_3_) spectrum of **AIZ-12-thymine.**

**
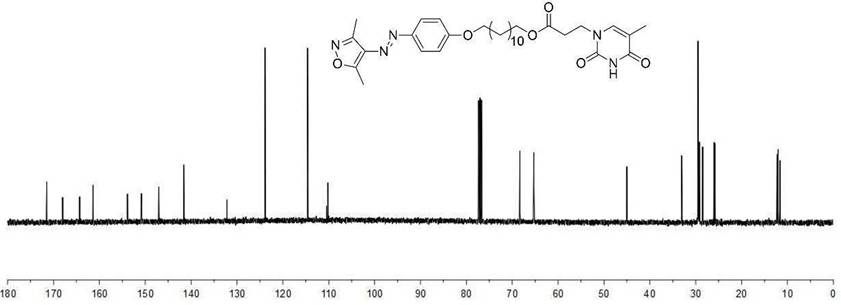
**

**Figure S7:** ^13^C-NMR (100 MHz, CDCl_3_) spectrum of **AIZ-12-thymine**.


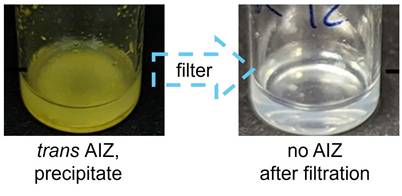


**Figure S8:** Photos of the dispersion of the mixture of the micelles and *trans*-AIZ-thymine before and after filtration.


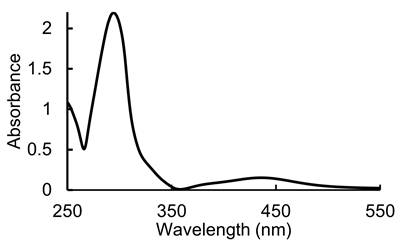


**Figure S9:** UV-vis spectrum of the dispersion after filtration of the mixture of the micelles and *cis*-AIZ-thymine.


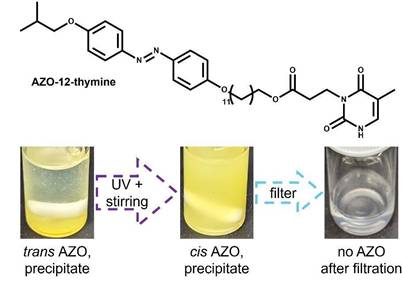


**Figure S10:** irradiation-filtration cycle on a conventional azobenzene-thymine derivative micelles dispersion resulting in a colorless dispersion not containing AZO.


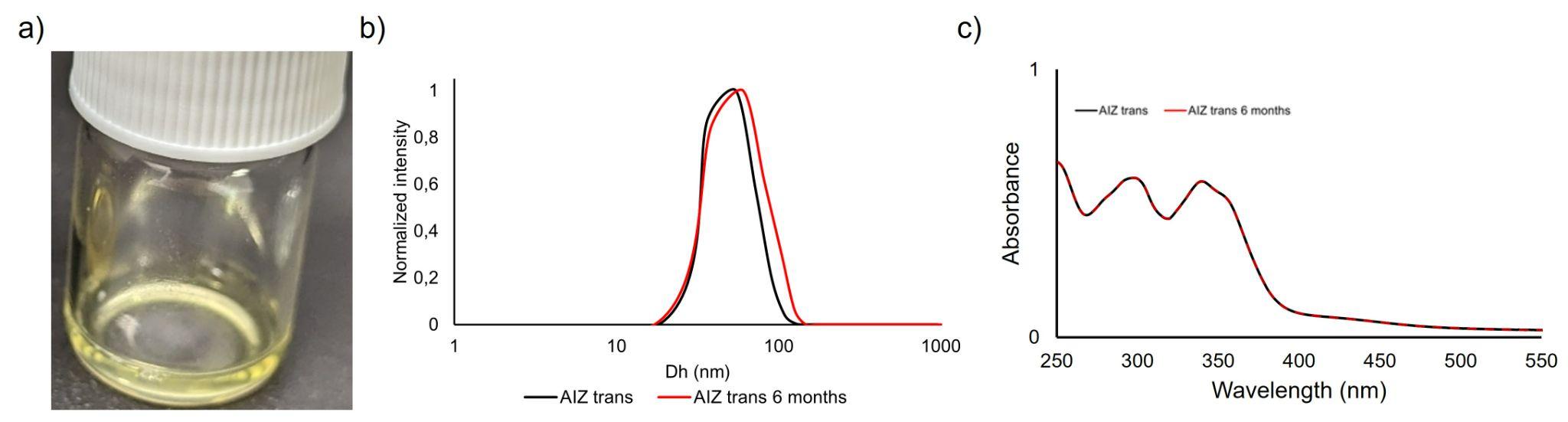


**Figure S11**: AIZ doped micelles after 6 months. a) photo of the AIZ micelles after 6 months, not showing precipitates; b) DLS traces (normalized in intensity, calculated on the average of 5 measures) not showing relevant differences after 6 months; c) UV-vis spectra of the AIZ micelles not showing differences in absorbance after 6 months.


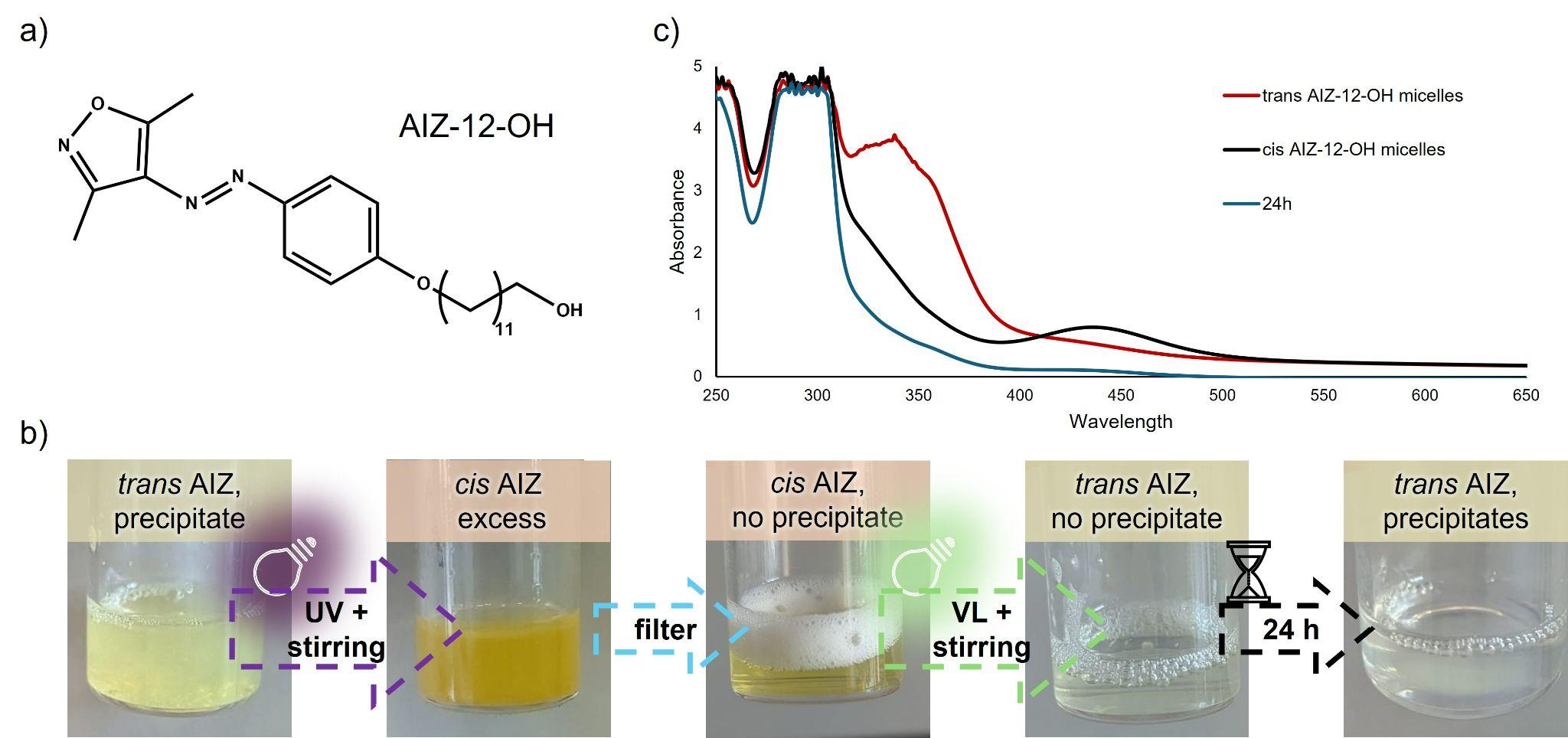


**Figure S12.** Incorporation and instability of the control photoswitch AIZ-12-OH. a) Molecular structure of the AIZ-12-OH photoswitch; b) Schematic representation and photographs of the light-mediated incorporation process: UV irradiation promotes the cis isomer, enabling transient micelle loading, while visible light regenerates the trans form. After 24 h, the photoswitch precipitates, indicating the absence of stable anchoring within the micellar core; c) UV–vis spectra of *trans*- and *cis*-AIZ-12-OH micelles, and the same dispersion after 24 h, showing loss of the characteristic AIZ absorption bands consistent with photoswitch release from the micelles.


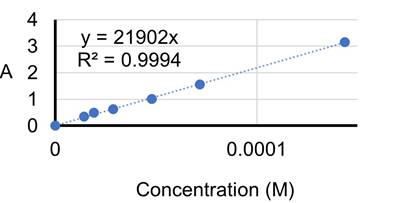


**Figure S13:** Calibration curve of absorbance at 338 nm versus **AIZ-12-thymine** concentration in methanol, used to quantify photoswitch loading in micelles.


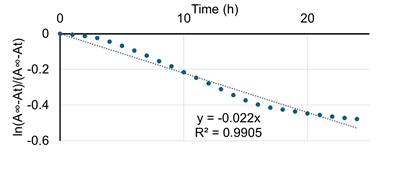


**Figure S14:** Relaxation process of **AIZ-12-thymine** *cis* isomer in methanol, showing a half-life time of 32 h.


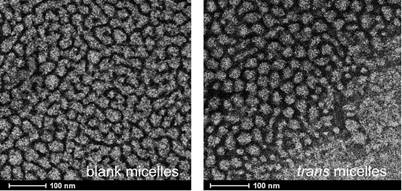


**Figure S15:** TEM images comparison between blank micelles (left image) and *trans* micelles (right image), not showing significant differences in morphology or dimensions.

**Table S1.** Pairwise post‑hoc comparisons of micelle diameter distributions unpaired t-test for comparisons between the different micelles populations (blank, *trans*, *cis* micelles).

| **Comparison** | ***p*** | **Significant** |
| --- | --- | --- |
| blank vs *cis* | 0.0295 | Yes |
| *blank* vs *trans* | 0.0007 | Yes |
| *cis* vs *trans* | 0.0001 | Yes |


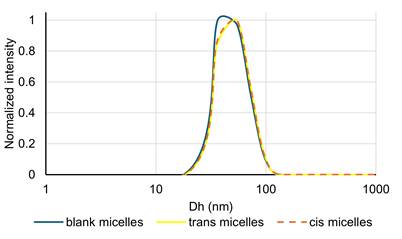


**Figure S16:** DLS traces of blank, *trans* and *cis* micelles obtained by the average of 5 measures for each population and normalizing the traces, not showing significant differences in *D_h_*.


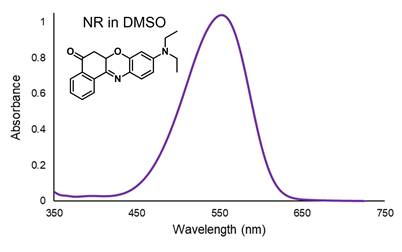


**Figure S17:** Absorption spectrum of free Nile Red in DMSO (λ_max_ ≈ 550 nm).


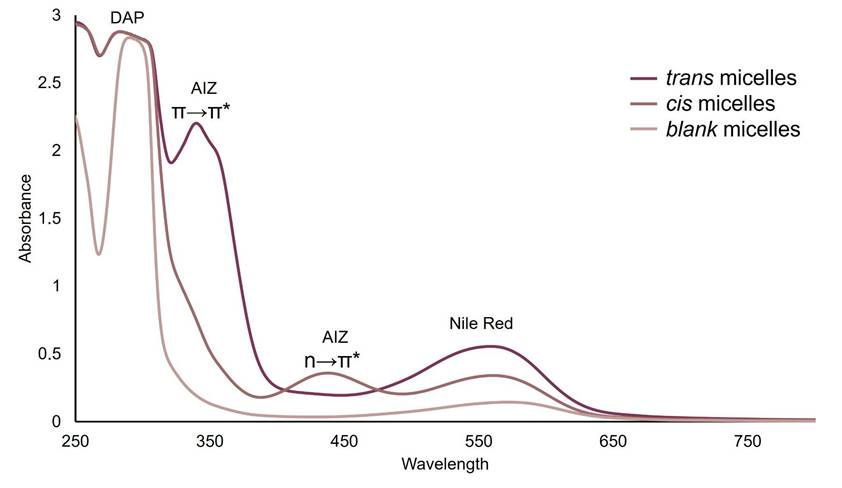


**Figure S18:** UV-vis spectra of blank, *trans* and *cis* micelles loaded with NR in a wavelength range of 250-750 nm.


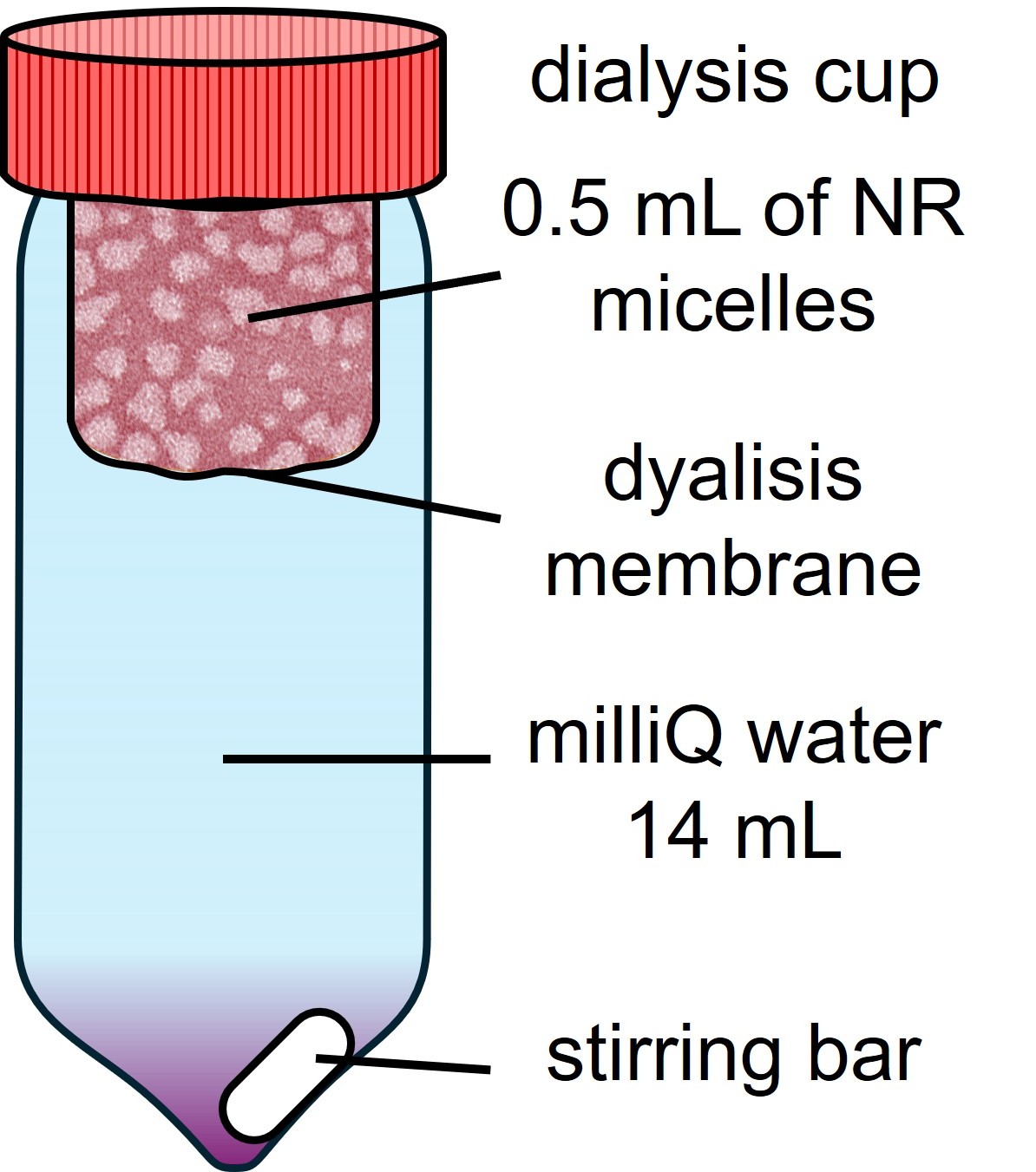


**Figure S19:** dialysis setup scheme: 0.5 mL of micelles loaded with Nile Red are put in a dialysis cup with a 3.5 kDa MWCO membrane, to perform a dialysis against 14 mL of Milli-Q® water at room temperature under magnetic stirring (200 rpm).


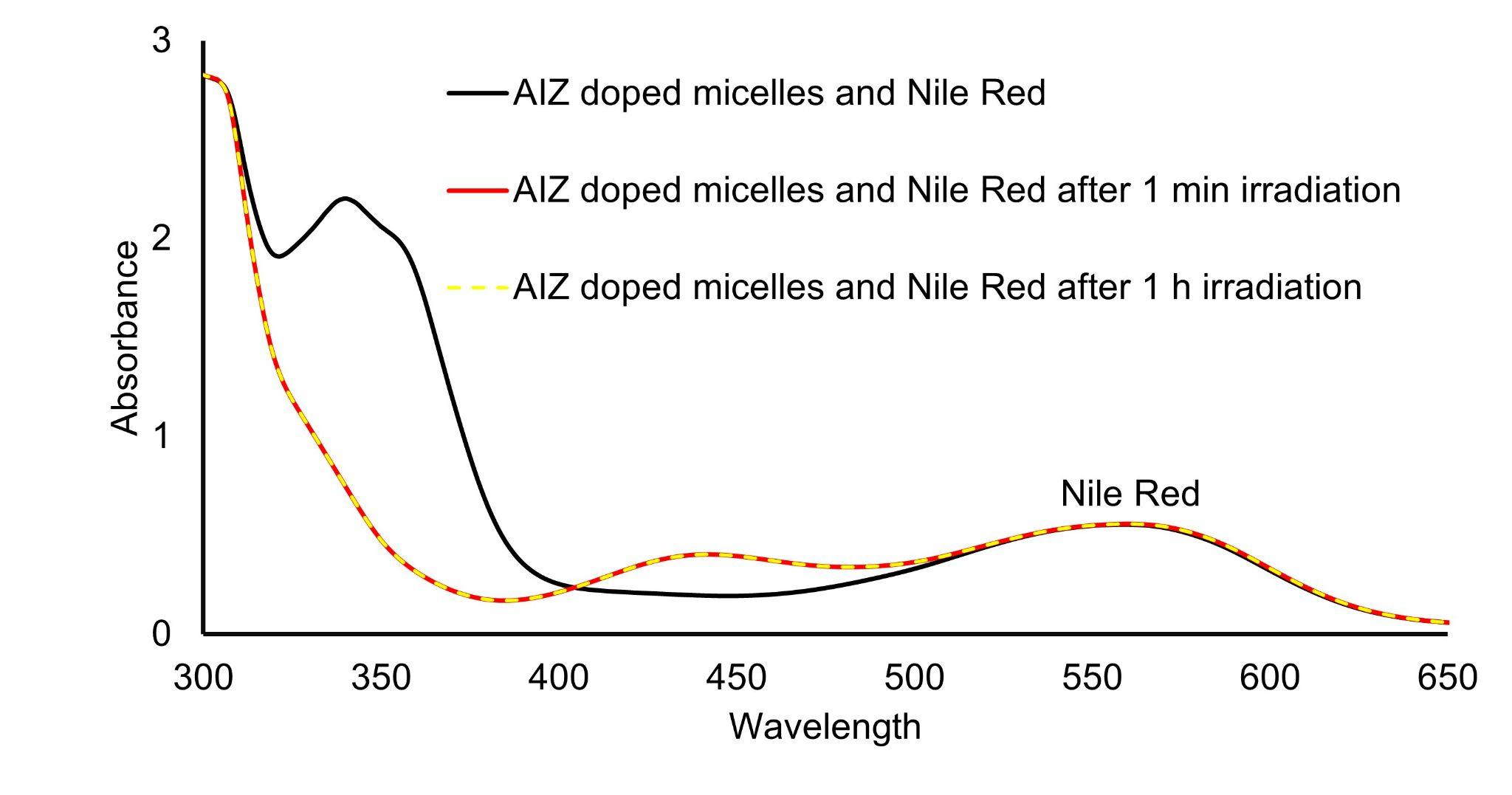

**Figure S20.** UV–vis spectra of AIZ-doped micelles containing Nile Red before and after 1 min and 1 h of irradiation at 365 nm (6.3 mW cm⁻²). The spectra show no variation in absorbance intensity or profile, confirming that Nile Red remains photostable under the experimental conditions used for micelle activation.


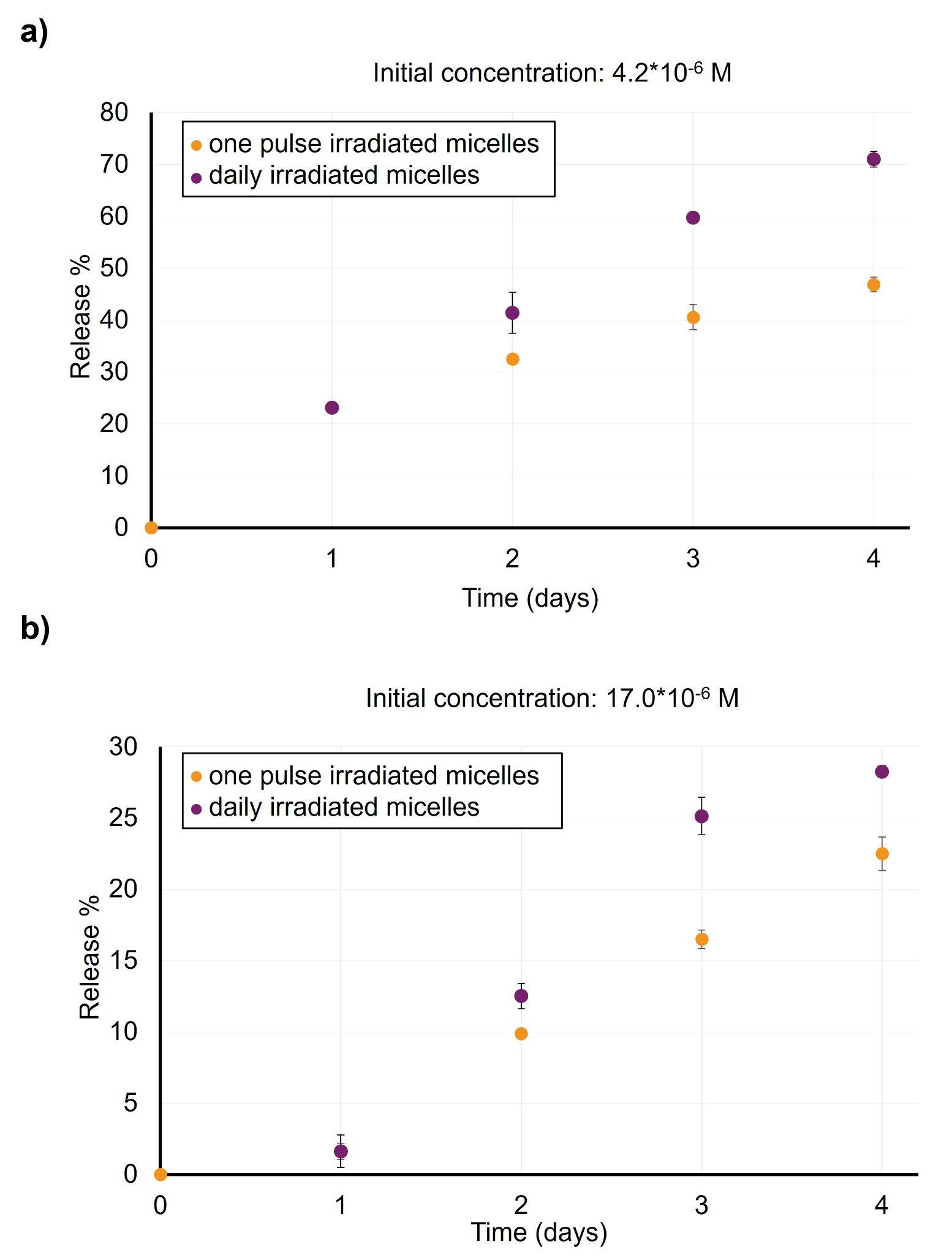


**Figure S21**: Release profiles of Nile Red on one pulse irradiated micelles (irradiated micelles in the main text) and daily irradiated micelles at initial loadings of a) 4.2×10⁻⁶ M and b) 17.0 × 10^-6^ M.
